# Continuous Aerobic Training in Individualized Intensity Avoids Spontaneous Physical Activity Decline and Improves MCT1 Expression in Oxidative Muscle of Swimming Rats

**DOI:** 10.3389/fphys.2016.00132

**Published:** 2016-04-18

**Authors:** Pedro P. M. Scariot, Fúlvia de Barros Manchado-Gobatto, Adriana S. Torsoni, Ivan G. M. dos Reis, Wladimir R. Beck, Claudio A. Gobatto

**Affiliations:** ^1^Laboratory of Applied Sport Physiology, School of Applied Sciences, University of CampinasLimeira, Brazil; ^2^Laboratory of Metabolic Disorders, School of Applied Sciences, University of CampinasLimeira, Brazil

**Keywords:** rodents, spontaneous physical activity, locomotor activity, sedentary behavior, aerobic training, monocarboxylate transporters (MCT), lactate, skeletal muscle

## Abstract

Although aerobic training has been shown to affect the lactate transport of skeletal muscle, there is no information concerning the effect of continuous aerobic training on spontaneous physical activity (SPA). Because every movement in daily life (i.e., SPA) is generated by skeletal muscle, we think that it is possible that an improvement of SPA could affect the physiological properties of muscle with regard to lactate transport. The aim of this study was to evaluate the effect of 12 weeks of continuous aerobic training in individualized intensity on SPA of rats and their gene expressions of monocarboxylate transporters (MCT) 1 and 4 in soleus (oxidative) and white gastrocnemius (glycolytic) muscles. We also analyzed the effect of continuous aerobic training on aerobic and anaerobic parameters using the lactate minimum test (LMT). Sixty-day-old rats were randomly divided into three groups: a baseline group in which rats were evaluated prior to initiation of the study; a control group (Co) in which rats were kept without any treatment during 12 weeks; and a chronic exercise group (Tr) in which rats swam for 40 min/day, 5 days/week at 80% of anaerobic threshold during 12 weeks. After the experimental period, SPA of rats was measured using a gravimetric method. Rats had their expression of MCTs determined by RT-PCR analysis. In essence, aerobic training is effective in maintaining SPA, but did not prevent the decline of aerobic capacity and anaerobic performance, leading us to propose that the decline of SPA is not fully attributed to a deterioration of physical properties. Changes in SPA were concomitant with changes in MCT1 expression in the soleus muscle of trained rats, suggestive of an additional adaptive response toward increased lactate clearance. This result is in line with our observation showing a better equilibrium on lactate production-remotion during the continuous exercise (LMT). We propose an approach to combat the decline of SPA of rats in their home cages. This new finding is worth for scientists who work with animal models to study the protective effects of exercise.

## Introduction

Lactate is not only a final product (waste) of glycolysis but also may operate as a metabolic signal, a pseudo-hormone (Brooks, 2002). Lactate is continuously produced by skeletal muscle at all levels of muscle activity (McDermott and Bonen, 1992), and it serves as an energy substrate for cardiac and skeletal muscles (Wilson et al., 1998; Pilegaard et al., 1999; Bonen, 2000). The release of lactate from the muscle into the blood and its uptake by other muscle cells occur via monocarboxylate transporters (MCTs), which are proton-linked membrane carriers (Juel and Halestrap, 1999; Gladden, 2000). Among the identified isoforms, MCT1 appears to be responsible for the influx of lactate predominantly in oxidative fibers, while MCT4 facilitates lactate extrusion mostly in glycolytic fibers (Wilson et al., 1998; Pilegaard et al., 1999; Bonen, 2000).

It has been shown that lactate transport, and consequently MCTs, can be altered in relation to the metabolic challenges placed on skeletal muscle by contractile activity (Baker et al., 1998; Enoki et al., 2006). In this context, aerobic training may increase lactate clearance in rats (Donovan and Brooks, 1983), mostly likely by increasing the ability of skeletal muscle to remove lactate from the blood (Baker et al., 1998). However, this topic is not fully resolved and other unexplored possibilities remain, as for example how the physical training at the maximal aerobic capacity intensity (i.e., anaerobic threshold) modulates these adaptations in predominantly red or white muscles.

Apart from metabolic context, it has been shown by Martins et al. (2004) that aerobic swimming exercise may also modulate the brain factors, which could influence the physical activity in daily life that is categorized as spontaneous physical activity (SPA; Garland et al., 2011; Kotz et al., 2012; Perez-Leighton et al., 2014). However, there are no studies available to show whether aerobic training could improve SPA of rats in their home cages (e.g., fidgeting, grooming, rearing, ambulatory locomotion).

Because every movement in daily life is generated by skeletal muscle, it is clear that an enhancement of SPA may lead to higher energy dissipation in this tissue (Gavini et al., 2014). Taking into consideration that skeletal muscles are both a consumer and a producer of lactate, we think that it is possible that an enhancement in SPA may be partly responsible for changes in lactate transport. Thus, we hypothesized that continuous aerobic training is capable to increase SPA of the rats, improve the expression of MCTs 1 and 4 in predominant oxidative and glycolytic muscles, and also should increase aerobic and anaerobic parameters. In order to test this, the aim of this study was to evaluate the effect of 12 weeks of continuous aerobic training in individualized intensity on SPA of rats and their gene expressions of MCT1 and MCT4 in soleus (oxidative) and white gastrocnemius (glycolytic) muscles. We also analyzed the effect of 12 weeks of continuous aerobic training on aerobic and anaerobic parameters using the lactate minimum test (LMT).

## Materials and methods

### Animals

Sixty-days-old male Wistar rats (*Rattus norvegicus albinos*), weighing 250–316 g at the beginning of the experiment and 388–544 g at the end. This work was previously approved by the institutional ethics committee (CEUA-UNICAMP, protocol: 2666-1). All experiments were conducted according to The National Institutes of Health's Guide for the Care and Use of Laboratory Animals. The same group of rats were kept together since weaning (21 days-old) on the controlled conditions. New rats (“intruders”) were not included in cages with already-established social relationships on any occasion. The animals were kept at room with controlled temperature (23 ± 1°C), relative humidity (45–55%), noise (< 80 decibels) and a photoperiod of 12:00 h light/dark cycle (lights switched on at 06:00 h). The rats had free access to water and food (chow Nuvilab®) at all times to prevent disturbance of social relationships. Rats were maintained in collective cages (5 rats/cage, 5331 cm3/rat, 49 cm of length, 34 cm of width, and 16 cm of height).

### Procedure

#### Experimental groups

In this study, animals (*n* = 30) were randomly allocated into three groups: a baseline group in which rats were evaluated and euthanized prior to start of the experimental training to characterize pre-training values for parameters studied; a control group (Co) in which rats were transported to the laboratory room, handled and experienced to water swimming stress throughout the study period; and training exercise group (Tr) in which rats were subjected to aerobic training for 12 weeks. More details on procedures are provided in the following methods sections. After the experimental period, SPA of rats was measured using a gravimetric method adapted from Biesiadecki et al. (1999). Aerobic and anaerobic parameters of animals were evaluated by LMT, and the expression of MCTs was determined by RT-PCR analysis.

#### Training schedule

Continuous aerobic training program lasted 12 weeks (5 days/week), and the rats swam for 40 min/day, with an overload equivalent to 80% of the anaerobic threshold, determined by the lactate minimum test (de Araujo et al., 2007). Exercise intensity was imposed upon the animals using elastics by attaching small lead weights (relative to the animal's body mass) to their upper back. Exercise sessions and laboratory procedures were always conducted at the same time of the day (08:00 a.m.). Water temperature was kept at 31 ± 1°C in all occasions (Harri and Kuusela, 1986). All swimming exercise bouts were conducted in cylindrical tanks (30 cm diameter × 120 cm depth). The use of tanks deeper than 100 cm with a smooth surface and individual compartments makes it impossible for the animals to rest at the tank bottom or jump to escape. In this regard, the continuous swimming behavior must be encouraged for swimming rats (Kregel et al., 2006), and this behavior was recurrent in our experiment, which indicated a good acclimatization of trained and untrained rodents to the swimming task. Fifteen days before starting the study, rodents were progressively allowed to swim to let them adapted to the handling and stress of the swimming task (Kregel et al., 2006). The untrained rats swam in deep water with elastics, but without weights attached to their thorax throughout the study period (5 min, 2 days/week). This kind of manipulation for the control group have been employed by us (de Araujo et al., 2013b) with the intention to mimic water swimming stress without promoting physiological adaptations confounding the effects of aerobic training.

#### Lactate minimum test (LMT)

Animals were subjected to the LMT (de Araujo et al., 2007) after the SPA measurement. The LMT determines an exercise intensity (known as lactate minimum intensity) that represents the maximal equilibrium between production and removal of blood lactate (i.e., anaerobic threshold). The lactate minimum intensity has been successfully determined in previous studies and show good relationship with the aerobic capacity (de Araujo et al., 2007, 2013b). The LMT consisted of three steps: (1) hyperlactatemia phase, (2) passive recovery, and (3) incremental exercise test. Hyperlactatemia (blood lactate increase) was induced by subjecting the animals to two short bouts of high-intensity exercise at 13% of the body mass (bm), separated by a 30 s passive recovery time. The first bout was 30 s in duration, and the second exercise bout lasted until exhaustion. The time to exhaustion during high-intensity exercise at 13% (2nd exercise bout) was recorded for each rat, and considered as an anaerobic parameter. After 9-min rest (passive recovery) to allow for the release of lactate from the exercised muscle into the bloodstream, blood samples were collected and, then animals were subjected to incremental exercise test. The incremental test began with an initial workload equal to 4% bm that increased by 0.5% bm every stage (4, 4.5, 5, 5.5, 6, and exceptionally 7% bm) until animals could not maintain the work required. The workload was gradually increased, and blood samples (25 μL) were collected at each 5 min. The lactate minimum intensity was achieved from the zero point derived from the second-order polynomial fit for the lowest lactate value of the “U-shaped” curve of blood lactate concentration versus load of the incremental phase of the test (de Araujo et al., 2007). The criteria for the success of test were the presence of the fit in the form of a “U” and the coefficient of determination (*R*^2^) of the polynomial fit greater than 0.75.

#### Spontaneous physical activity measurement

The experimental rats were placed in their cages to allow them to become acclimated to the environment, and then subjected to SPA measurement beginning at least 24 h after the last training session. SPA measurement was undetectable to the rats. At the same time, the control rats had their SPA measured in the absence of any treatment. On the day of testing, rats were weighted. Considering that social animal organization stabilizes and becomes subtle when adequate conditions in which animals live are preserved (Olfert et al., 1993); and given that the disruptions of social organization were negligible in our experiment, we believe that any modification in the movements of well-acclimated rats in collective cages is a sign of individual changes rather than social housing changes. Taking into account that social isolation can be distressing to Wistar rats (Wiberg and Grice, 1963; Brain and Benton, 1979; Gil et al., 1999; Sharp et al., 2002), the SPA measurements were conducted with social housing (five rats per cage) instead of providing one cage for a single animal. All movements of rats, in their cages (two cages *per* group), were registered by the system as changes in weight and continuously recorded for 20 h period (10/10 h dark/light period). We classified one whole day into two periods: dark period (20:00 p.m. to 05:59 a.m.) and light period (06:00 a.m. to 16:00 p.m.). The 4 h period was eliminated from analysis to remove disturbances related to human access.

#### Apparatus to SPA determination

**Figure 1:**
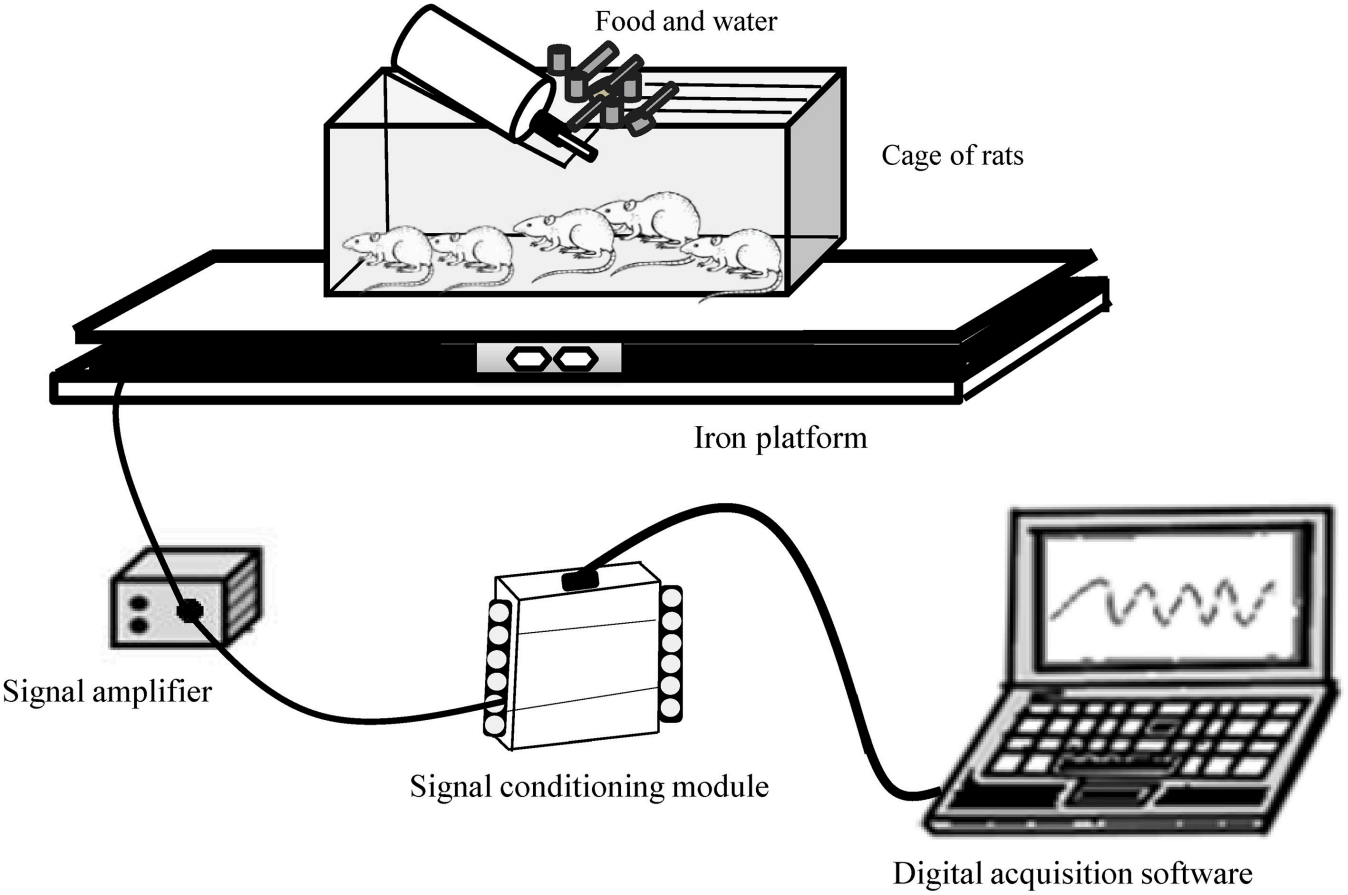
**Gravimetric apparatus for measuring spontaneous physical activity of rats**.

SPA of rats was measured using a gravimetric method adapted from Biesiadecki et al. (1999). As illustrated in Figure 1, animal's cages was placed on two iron platforms (47 × 40 cm) where was fixed between them a high sensitive load cell (primary sensor, PLA30Kgf®, Lider Balanças™). The output from the load cell was fed into a signal amplifier (MKTC5-10®, MK control and instrumentation™) and then to an analogic to digital board converter (USB-6008® signal-conditioning module) in a computer where it was collected at a frequency of 30 Hz using software developed from the LabView package (Signal Express® 2009, National Instruments™). The signal acquisition system was calibrated by applying known mass. Regression equations (*R*^2^ = 0.99) were then computed enabling conversions of milivolts (mv) signals to kilograms (kg) units. Biesiadecki et al. (1999) and other authors (Chausse et al., 2014; Moes and Holden, 2014; Beck et al., 2016) have shown the efficacy of gravimetric principle. Our apparatus was designed to be operated without human presence. Because it was not necessary to remove the rats from their own keeping cage in the biotery, it was possible to measure their natural SPA. This technique can provide accurate information about the animal's behavior.

#### Euthanasia

At 150-days-old, 48 h after LMT, the rats in rest condition, were anesthetized using 50 mg/kg sodium thiopental. After the loss of cornea and foot reflexes, the animals were euthanized, using the decapitation method. The soleus (oxidative) and white gastrocnemius (glycolytic) muscles were dissected, immediately frozen in liquid nitrogen and stored at −80°C.

### Data analysis

#### Spontaneous physical activity treatment

After data acquisition, the raw data were processed in the Butterworth digital filter of 4th order using MATLAB®7.0 software (MathWorks™). SPA was achieved by the difference values among each consecutive samples (*Cs*) which were squared, taken the square root and summed to each hour. After, data were adjusted by dividing the SPA values by the body mass (grams) of rats of each cage (kg × g^−1^). The SPA equation is defined as, according Chausse et al. (2014):

$$\begin{array}{l} SPA\;=\;\overset{n}{\underset{i=1}{\sum }}\sqrt{{\left({Cs}_{i\;+\;1}\;-\;{Cs}_{i}\right)}^{2}} \end{array}$$

#### Blood lactate analyses

During LMT, blood samples (25 μL) were collected from the rats' distal tails with a capillary tube that had been previously calibrated. The blood was transferred to tubes (1.5 mL) containing 400 μL of trichloroacetic acid (C_2_HCl_3_O_2_) at [4%]. The samples were immediately stored at a temperature between 2 and 8°C. Blood lactate concentration analyses were carried out on a microplate reader (ASYS Expert Plus UV, Biochrom) by enzymatic method as previously described (Engel and Jones, 1978). The absorbances were measured at 340 nm and were normalized against a calibration curve.

#### Quantitative real-time PCR (RT-PCR analysis)

Total RNA was extracted from the white gastrocnemius and soleus muscles using Trizol reagent (Life Technologies Corporation, CA, USA) according to the manufacturer's recommendations. Total RNA was quantified using a Nanodrop ND-2000 (Thermo Scientific, WI, USA). Reverse transcription was performed with 3 μg of total RNA using a High-Capacity cDNA Reverse Transcription kit (Life Technologies Corporation, California, USA). The messenger RNA (mRNA) was measured using primers and a TaqMan detection system obtained from Applied Biosystems: MCT-1 (Slc16a1, GenBank accession NM_012716) and MCT-4 (Slc16a3, GenBank accession NM_030834). Glyceraldehyde-3-phosphate dehydrogenase primer (Applied Biosystems, cat n# 4352338E) was used as an endogenous control. Real-time PCR analysis of the gene expression was performed on an ABI Prism 7500 Fast platform using 20 ng of cDNA. The data were analyzed using a Sequence Detection System 2.0.5 (Life Technologies Corporation, CA, USA) using the comparative threshold cycle method (2^−ΔΔ^Ct), according to the manufacturer's recommendation.

#### Statistical procedures

Statistical analyses were carried out using a software package (Statistic 7.0). Data normality was checked using the Shapiro-Wilk. Data are presented in means and standard error of the mean (SEM). For comparisons among groups, two-way ANOVA (time × training) was employed. Newman-Keuls post hoc test was used to locate group's difference. It was also calculated the effect size (Cohen's d) for the studied variables. Effect size was determined by the formula: (mean1 – mean2)/pooled standard deviation. The thresholds for small, moderate, and large effects were 0.20, 0.50, and 0.80, respectively. The significance level was set at *P* < 0.05 in all cases.

## Results

There were no significant differences in SPA among groups during light period (Table 1). However, in dark period, SPA decreased from baseline to 12 weeks in the Co group (*P* = 0.04; effect size = 0.81), but not in the Tr group (*P* = 0.51; effect size = 0.37; Table 1). No statistical difference was found in the SPA (dark period) between the Co and Tr groups (*P* = 0.18; effect size = 0.71). Body mass increased from baseline to 12 weeks in the Co group (*P* = 0.00; effect size = 6.46) and Tr group (*P* = 0.00; effect size = 5.80). Moreover, Co group shows a greater body mass than Tr group (*P* = 0.03; effect size = 0.84; Table 1).

**Table 1:** **Spontaneous physical activity (SPA) during dark and light periods, and body mass for the baseline, control (Co) and training exercise (Tr) groups**.

	**0 week**	**12 weeks**	
	**Baseline**	**Co**	**Tr**
SPA—Dark period (kg × g^−1^)	0.298 ± 0.030	0.219 ± 0.014^*^	0.262 ± 0.013
Δ% in relation to baseline		–26.5	–12.0
SPA—Light period (kg × g^−1^)	0.072 ± 0.016	0.040 ± 0.004	0.038 ± 0.004
Δ% in relation to baseline		–43.9	–47.0
Body mass (g)	288.27 ± 6.32	490.41 ± 14.53^*^^#^	455.83 ± 14.68^*^
Δ% in relation to baseline		70.1	58.1

*Percent of variation (Δ) in relation to baseline group*.

*For both periods, SPA data (Mean ± SEM) are 10 h values for each cage (2 cages per group), totalizing n = 20. Sig. diff*.

* P < 0.05 vs. baseline group;

# *P < 0.05 vs. Tr group. Newman-Keuls post-hoc test was used to locate group's difference*.

**Figure 2:**
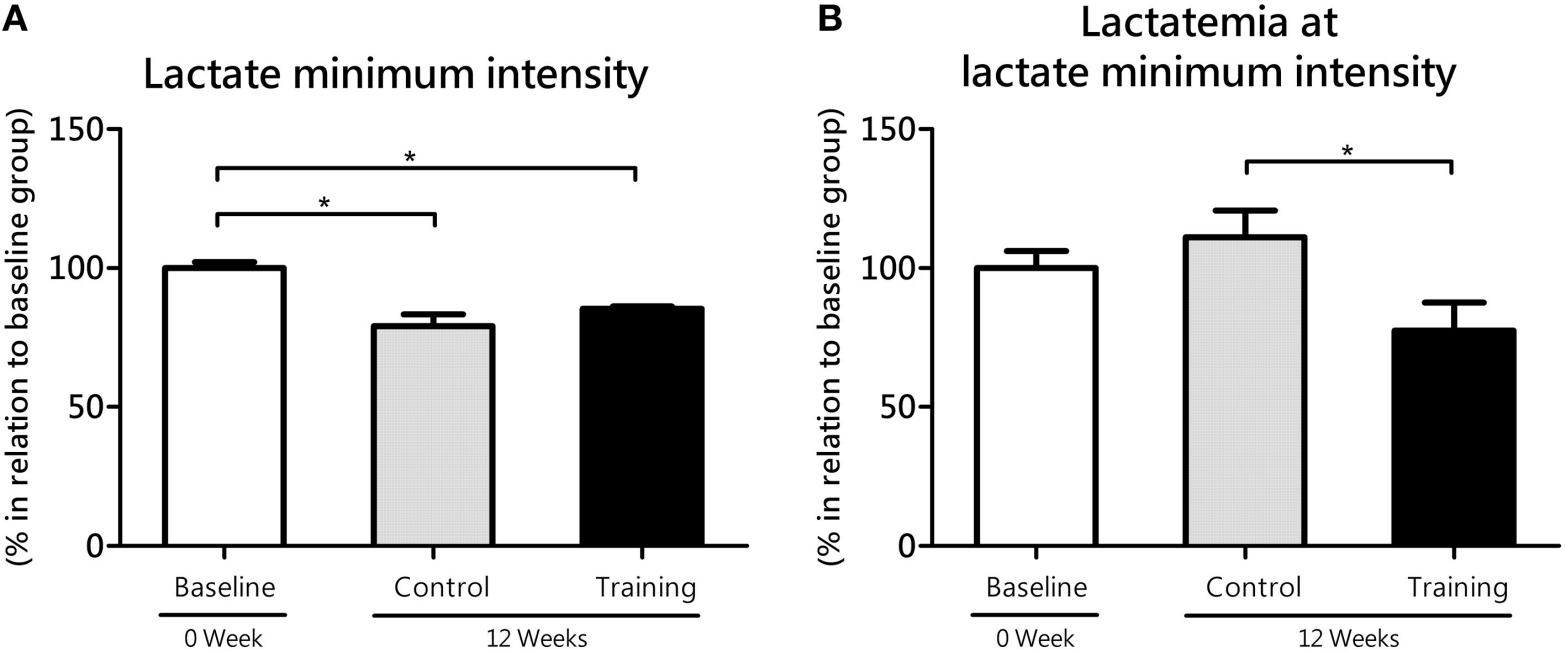
**The lactate minimum intensity (A) relative to the animal's body mass (aerobic parameter) and blood lactate levels (lactatemia) at this intensity (B) for the control and training exercise groups**. The data (mean ± SEM) are relative (%) to baseline group, which has been set at 100%. Newman-Keuls *post-hoc* test was used to locate group's difference. *N* = 7–10 animals per group. Sig. diff. ^*^P < 0.05.

No statistical difference (*P* = 0.11; effect size = 1.32) was found in the lactate minimum intensity between the Co and Tr groups; however, Co group (*P* = 0.00; effect size = 2.90) and Tr group (*P* = 0.00; effect size = 3.19) showed lower values for lactate minimum intensity than baseline group (Figure 2A). The lactate minimum intensity relative to the animal's body mass was correspondent to 5.16 ± 0.12, 4.07 ± 0.22, and 4.40 ± 0.04% bm to the baseline, Co and Tr groups, respectively. As presented in Figure 2B, Tr group showed significantly lower lactatemia at lactate minimum intensity than Co group (*P* = 0.04; effect size = 1.72), demonstrating that lactate accumulation in blood is reduced after a period of aerobic training. There is no statistical difference in lactatemia at lactate minimum intensity between the baseline group compared to the Co group (*P* = 0.36; effect size = 0.61) and Tr group (*P* = 0.17; effect size = 1.07). The blood lactate concentration at lactate minimum intensity was correspondent to 6.86 ± 0.43, 7.62 ± 0.66, and 5.32 ± 0.68 mmol L^−1^ to the baseline, Co and Tr groups, respectively.

**Figure 3:**
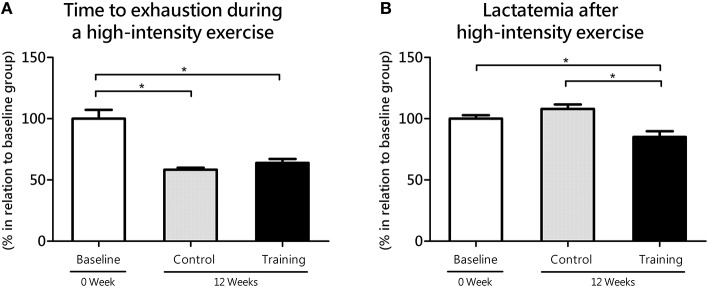
**Time to exhaustion during high-intensity exercise (anaerobic parameter, 2nd bout of hyperlactatemia induction at the lactate mininum test; A) and its responses on blood lactate levels (lactatemia after 9 min of the anaerobic exercise; B) for the control and training exercise groups**. The data (mean ± SEM) are relative (%) to baseline group, which has been set at 100%. Newman-Keuls *post-hoc* test was used to locate group's difference. *N* = 8 –10 animals per group. Sig. diff. ^*^p < 0.05.

Regarding to the time to exhaustion during high-intensity exercise, no statistical differences (*P* = 0.44; effect size = 0.79) were found between the Co and Tr groups; however Co group (*P* = 0.00; effect size = 3.01) and Tr group (*P* = 0.00; effect size = 2.02) showed lower time to exhaustion than baseline group (Figure 3A). The time to exhaustion during high-intensity exercise was correspondent to 91.8 ± 6.6, 53.5 ± 1.6, and 59.6 ± 3.9 s to the baseline, Co and Tr groups, respectively. After 9-min rest of the high-intensity exercise, the blood lactate concentration was correspondent to 6.89 ± 0.19, 7.43 ± 0.25, and 5.85 ± 0.32 mmol L^−1^ to the baseline, Co and Tr groups, respectively. As exposed in the Figure 3B, trained rats showed lower lactatemia after 9 min of the anaerobic exercise compared to the Co group (*P* = 0.00; effect size = 1.97) and the baseline group (*P* = 0.01; effect size = 1.42). There is no statistical difference in lactatemia after 9 min of the anaerobic exercise between the baseline and Co groups (*P* = 0.10; effect size = 0.80).

**Figure 4:**
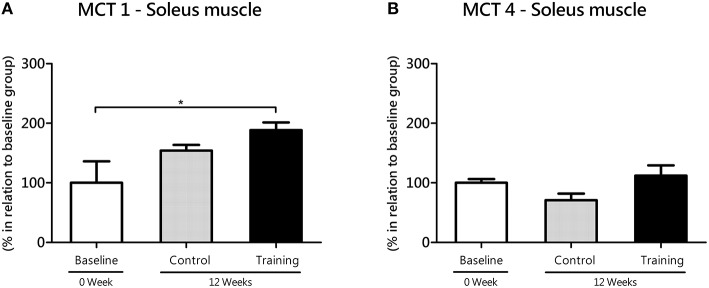
**mRNA level of monocarboxylate transporters (MCT) 1 (A) and 4 (B) in the soleus (oxidative) muscle for the control and training exercise groups**. The data (mean ± SEM) are relative (%) to baseline group, which has been set at 100%. Newman-Keuls post hoc test was used to locate group's difference. *N* = 7–10 rats per group. Sig. diff. ^*^P < 0.05.

In soleus (oxidative) muscle, MCT1 increased from baseline to 12 weeks in the Tr group (*P* = 0.04; effect size = 1.53), but not in the Co group (*P* = 0.12; effect size = 1.05; Figure 4A). Despite of the lack of difference between Co and Tr groups (*P* = 0.32), a large effect size (1.17) was found. As exposed in the Figure 4B, there is no statistical differences in MCT4 mRNA level (soleus) among three groups studied. In spite of the lack of difference between Co and Tr groups (*P* = 0.37), a large effect size (1.10) was found.

**Figure 5:**
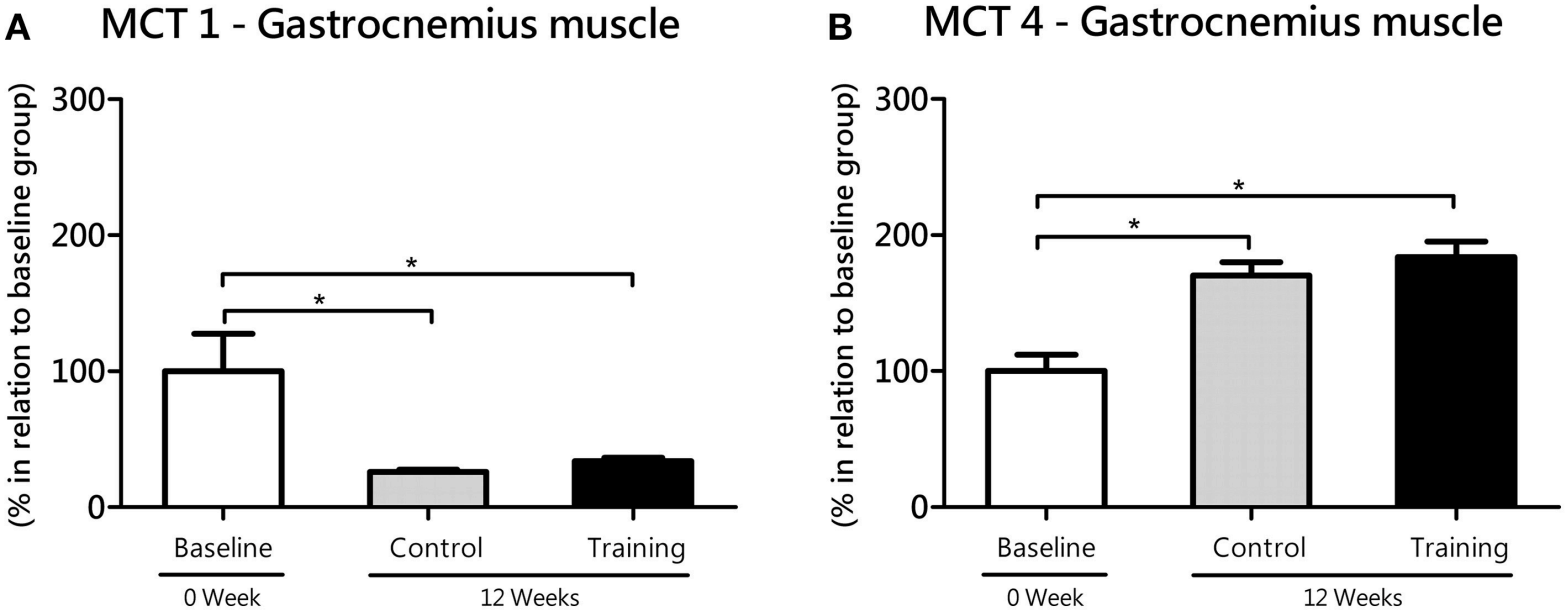
**mRNA level of monocarboxylate transporters (MCT) 1 (A) and 4 (B) in the white gastrocnemius (glycolytic) muscle for the control and training exercise groups**. The data (mean ± SEM) are relative (%) to baseline group, which has been set at 100%. Newman-Keuls post hoc test was used to locate group's difference. *N* = 9 –10 rats per group. Sig. diff. ^*^P < 0.05.

In white gastrocnemius (glycolytic) muscle, no statistical differences were found between the Co and Tr groups with regard to MCT1 (*P* = 0.77; effect size = 1.50) and MCT4 (*P* = 0.40; effect size = 0.44). However, Co group (*P* = 0.04; effect size = 1.78) and Tr group (*P* = 0.03; effect size = 1.58) showed reduced gene expression of MCT1 compared with the baseline group (Figure 5A). According to ANOVA, this time-dependent effect was not affected by training, in agreement with the lack of significant time × training interaction (*F* = 0.04; *P* = 0.84). In opposition to MCT1, Co group (*P* = 0.00; effect size = 2.14) and Tr group (*P* = 0.00; effect size = 2.45) showed increased gene expression of MCT4 (gastrocnemius) as compared with the baseline group (Figure 5B), and this time-dependent effect was also not influenced by aerobic training since there is no significant interaction between time and training (*F* = 0.35; *P* = 0.55).

## Discussion

The main discovery of this study is related to protective effect of continuous aerobic training on decline of SPA. We found that SPA (dark period) decreased significantly in the control (Co) group after 12 weeks of experiment (Table 1). Does this finding reflect the unhealthy housing conditions in which rats live? One way to answer this question is to look for their common habits of daily life. In the wild, rodents have abundant opportunity to move and to explore their environment. Such is not the case when rodents are maintained in captivity in the most laboratories around the world. Recently, efforts in our group have focused on the captive management systems (Scariot et al., 2015). We believe that when we put animals in a small cage, in true, we submitted the animals to a compulsory sedentary status and, of course, and the adaptations (gene expression, remodeling tissues, etc.) are quite different than animals in natural environment. Thus, the control groups, normally referred in the literature, are far from a natural status. In particular, this issue incites a reflection about the way the scientist maintains the animals in the laboratory. Our assumption is that control rodents underwent a considerable amount of sedentary behaviors, resulting in a decrease of SPA. Now, would this be true even for trained rats? We admit we were almost sure this answer would be “yes” because of the similarity of the unhealthy environment in which rats live. Despite of this, we found that SPA (dark period) levels did not decrease in the trained rats after 12 weeks of experiment, which is new finding, and it is worth for scientists who work with animal models to study the protective effects of exercise.

Because SPA of rats in their home cages (e.g., fidgeting, grooming, rearing, ambulatory locomotion) can be analogous to aerobic exercise, we realize that trained rats would exhibit an enhanced skeletal muscle oxidative metabolism. This should be connected to an improvement in MCT1 in the soleus muscle, which is metabolically suited for lactate oxidation (Gladden, 2000). To test this idea, we evaluated the gene transcription of MCTs, which provide a sensitive indication for training-specific muscular adjustments with exercise training (Zoll et al., 2006). Here, we showed that MCT1 expression increased from baseline to 12 weeks in the soleus muscle of trained rats (Figure 4A), suggesting an improvement in the capacity of muscles to uptake lactate from blood. This suggestion is in line with our observation showing lower accumulation of blood lactate in trained compared to untrained rats when subjected to exercise testing (Figures 2B, 3B). For many years, different mechanisms has been proposed to explain how the aerobic training would affect lactate dynamics (Mole et al., 1971; York et al., 1974; Ivy et al., 1980; Donovan and Brooks, 1983; Holloszy and Coyle, 1984; Favier et al., 1986). Now, based on our results, it is very likely that the an enhanced SPA among aerobic-trained rats from a continuous exercise may also contribute to an increased lactate clearance, at least in a molecular aspects, despite of the similar results of lactate minimum intensity (i.e., anaerobic threshold) between Co and Tr groups.

The increase in MCT1 expression in the soleus of trained rats parallels the reduction in MCT1 expression in the white gastrocnemius muscle during the same experimental period, lead us to think that changes in lactate transport elicited by continuous aerobic training are more pronounced in oxidative than glycolytic muscles. This is in agreement with other published reports, which have indicated that oxidative muscles are more responsive to aerobic training than glycolytic muscles (Holloszy, 1976). Although, MCT1 lower expression in white muscles (Bonen et al., 2000), it may have an additional impact on aerobic exercise performance, which was never proved experimentally. Therefore, it is reasonable consider that the MCT1 expression reduction in glycolytic muscles found in this study may be a possible factor contributing to the decline in aerobic capacity with age, as demonstrated here (Figure 2A). Continuous aerobic training did not cause significant alterations in MCT4 expression of glycolytic muscles (Figure 5B). This result is coherent if we consider that, because of training settings (prioritizing aerobic gains), aerobic-trained rats does not overly depend upon engagement of the glycolytic pathway to energy production, what may be too different to the high intensity interval training for example. These aspects will be tested in future works, using controlled exercise loads, especially in anaerobic efforts.

We also examined the effects of the continuous aerobic training on aerobic and anaerobic parameters. For this purpose, we used the LMT, which has been successfully used by us (de Araujo et al., 2007, 2013b) to determines especially an exercise intensity that represents the maximal equilibrium between production and removal of blood lactate. As already reported by de Araujo et al. (2007), the lactate minimum intensity is linked to the aerobic capacity, which, in turn, is associated with a risk of metabolic diseases (Novak et al., 2010; Gavini et al., 2014). Our data suggest that the continuous aerobic training was effective in maintaining SPA, but did not prevent the decline of aerobic capacity (Figure 2A) and anaerobic performance (Figure 3A), leading us to consider that the decline of SPA common with age is not fully attributed to a deterioration of physical properties. Apart from metabolic context, the literature suggests that the diminished SPA associated with aging could be explained by compromised orexin signaling in the hypothalamus in animal models (Zink et al., 2014). The age range of our rats was of 2–5 months, which it can consider as young rats. However, although the most physiological changes on age are observed when it compares early age with the end in a life span, Feng et al. (2008) compared the orexin A levels between juvenile (35-days old) and adult (3–4 months old) rats and reported levels 10 times upper in younger animals. Although, of the orexin measurement techniques used by authors had been different among ages, and the different ages if compared with the present study, these specific orexin related aspects on SPA responses will be better investigated in our next studies, involving similar protocol.

One issue to be discussed is the reliability of our SPA data and the accuracy of our interpretation. Some authors have assumed (incorrectly) that regular exercise affects SPA of rodents, however their data come from open-field model (Skalicky et al., 1996; Viidik and Skalicky, 1997). In this model, animals are removed from their home cage, and then placed in a new environment. The open-field model has been employed to evaluate emotional (Broadhurst, 1957, 1958) and anxiety behavior (Angrini et al., 1998); however, the extent to which behavior in the open field correlates with SPA in a home cage is controversial (Walsh and Cummins, 1976). To overcome these issues, we adopted the gravimetric apparatus because of its consistency and good accuracy in measuring both large and small motor movements of animals in their own laboratory environment. These movements are, in fact, categorized as SPA (Biesiadecki et al., 1999). In addition, our apparatus enables to record natural behaviors (i.e., “pure” SPA) because it is imperceptible to the rats, and the human presence is unnecessary. This highlights one advantage of our method because it has been supposed that the behavior of animals may either be constrained or reinforced by the perception of the animals to the environment (Hirsjärvi and Junnila, 1986) or human contact (Hirsjärvi and Väliaho, 1995). Finally, we believe that an measurement of the movements performed instinctively by the animals (i.e., SPA) have great interest for physiologists and, it would also be of interest for behavioral researchers to further examine, for example, the social environment (Evans et al., 1994), drug self-administration (Weiss et al., 2003), deprivation effects (Lamas and Pellon, 1995), habituation, and sensitization in responsiveness to a stimulus (Aoyama and McSweeney, 2001) and impulsive behavior in attention deficit hyperactivity disorder (Garcia and Kirkpatrick, 2013).

Many studies focused only on exercise itself while ignoring the physical activity outside the exercise sessions (i.e., SPA). Studies support the idea that chronic exercise may affects SPA. For instance, Kriemler et al. (1999) noted that the exercise sessions “invigorated” the obese adolescents, who chose to increase activities during the non-training hours. Moreover, in a recent study, students who were not engaged in team sports were more likely to have sedentary behavior during school breaks (Silva and dos Santos Silva, 2015). Thus, the challenge for physiologists is to give appropriate attention to SPA; this variable should be considered with caution for a better understanding of biological systems in different experimental models. For example, it is entirely possible that overtraining caused by a dis-balance in training load and recovery (Pereira et al., 2012) may have adverse effects on SPA, beyond the obvious evidence of muscle-micro trauma, muscle soreness, inflammation, fatigue, and other functional deficits (Pereira et al., 2014). Moreover, we believe that, it is likely that higher SPA resulted from the continuous aerobic training could further stimulate energy expenditure after the exercise stimulus, which is termed as excess post-exercise oxygen consumption (EPOC; Borsheim and Bahr, 2003; LaForgia et al., 2006). In the final example, we highlight the excellent study made by Wang et al. (2003), who found increased expression of MCT1 and 4 in rats following 7 days of thyroid hormone (T3) ingestion. However, in this experimental protocol, authors had not considered the SPA status of the T3-treated animals in their analyses. Thus, the lactate transport improvement in the T3-treated rats found by these authors may have resulted from an increase in SPA since that hyperthyroidism is associated with this effect (Levine et al., 2003). Future studies are expected to explore questions raised in the above discussion.

To the best of our knowledge, this is the first study to investigate the effects of aerobic training at individualized intensity of LMT on SPA, MCTs expression and aerobic/anaerobic performance in rats. The continuous training prescription based on 80% of the anaerobic threshold (individualized for each rat) was intended to provide an intensity that would stimulating aerobic gains (de Araujo et al., 2013a). The lack of successful aerobic adaptations with regard to lactate minimum intensity does not represent an inefficiency of our training protocol. In line with our initial intuition, we strongly believe that living time in small cage may inhibit the benefits of aerobic training (Scariot et al., 2015). Despite attempts to train rats with exercise protocols (as example 40 min/day), the exercise time per day is relatively low (2.8%) when compared to time per day (97.2%) in which rats are confined in their home cages. This argument also may explain the lack of pronounced changes of MCTs between the Co and Tr groups in all muscles studied. As a result, future studies will be required to examine whether marked increases in MCT1 and MCT4 could be achieved because of the association among continuous and intermittent aerobic training and wide housing space.

It is important be mentioned that the present study was not designed to identify an optimal training intensity that would induce an increase in SPA, and this effect may be dependent on varying degrees of physical fitness. Despite of this, our study adds knowledge regard to a new benefit of the continuous aerobic training as a promising and safe strategy for attenuating the decline of SPA, once it is not necessary the use of loads involving higher exercise intensities. This strategy is greatly desired to avoid sedentary behaviors, and adverse health outcomes in animals models of many biological disciplines. It is well-known that low-SPA rats are susceptible to fat deposition (Teske et al., 2012) and body mass gain (Teske et al., 2006, 2014). Therefore, because of the great value of our observations, it is clear that the further analyses are essential to determine the relationship between aerobic training and SPA by examining key brain areas and neurotransmitters involved in energy balance.

## Conclusion

The continuous aerobic training is effective to prevent the decrease in SPA, but did not prevent the decline of aerobic capacity and anaerobic performance of *Wistar* rats collectively housed from 2 to 5 months. Moreover, we found a better equilibrium on lactate production-remotion during the continuous exercise. Changes in SPA were concomitant with changes in MCT1 expression in the soleus muscle of trained rats. Taken together, the results suggested that the unknown mechanism of enhanced SPA provided of the continuous aerobic training at 80% LMT may also contribute to an improvement in the capacity of muscles to uptake lactate from blood. Considering only SPA data, aerobic training may be a promising strategy in combating unhealthy behaviors of laboratory rats.

## Author contributions

All authors were responsible for data collection, figures, and table preparation and manuscript writing. In particular, Gobatto, C. A. was responsible for research funding, data analysis, manuscript/experiment design, and final review.

### Conflict of interest statement

The authors declare that the research was conducted in the absence of any commercial or financial relationships that could be construed as a potential conflict of interest.
